# Three‐dimensional echocardiography to identify right ventricular dilatation in patients with corrected Fallot anomaly or pulmonary stenosis

**DOI:** 10.1111/cpf.12665

**Published:** 2020-10-23

**Authors:** Aleksandra Trzebiatowska‐Krzynska, Eva Swahn, Lars Wallby, Niels Erik Nielsen, Carl Johan Carlhäll, Jan Engvall

**Affiliations:** ^1^ Department of Cardiology and Department of Medicine and Health Sciences Linkoping University Linkoping Sweden; ^2^ Department of Clinical Physiology and Department of Medicine and Health Sciences Linkoping University Linkoping Sweden; ^3^ CMIV – Center for Medical Image Science and Visualization Linkoping University Linkoping Sweden

**Keywords:** 2Dimensional echocardiography, 3Dimensional echocardiography, cardiac magnetic resonance, congenital heart disease, deformation imaging, exercise capacity, right ventricle

## Abstract

**Background:**

3‐Dimensional Echocardiography allows measuring volumes and parameters of myocardial deformation (strain). Myocardial strain has been suggested to be superior to conventional echo parameters in the assessment of right ventricular (RV) function. Myocardial strain can be assessed by cardiac magnetic resonance (CMR) or two‐ and three‐dimensional echocardiography (2D and 3DEcho). We performed a comprehensive assessment of the RV based on 3DEcho and compared the results with those based on CMR and 2DEcho.

**Methods:**

36 patients with corrected heart defects underwent CMR and 3DEcho to assess RV volume, strain and cardio pulmonary exercise testing with peak VO_2_ measurement. 2DEcho was used for reference.

**Results:**

There was a moderate correlation between 3DEcho and CMR for measuring RV end‐diastolic and end‐systolic volumes (r = .82 and .72). 3DEcho tended to underestimate the RV volumes, mean difference EDV 8.5 ± 33 ml (CI −2.8; 19.7 ml) and ESV 13.2 ± 29 ml (CI 3.3; 23 ml). According to method‐specific reference values for RVEDV, 34/35 (3DEcho) and 29/36 (CMR) were dilated. Among those dilated according to CMR, all were identified by 3DEcho. The coefficient of correlation between RV atrioventricular plane displacement measured by CMR and tricuspid annular plane systolic excursion measured by 3D and 2DEcho was r = .6 for both. 2DEcho measured lower LV volumes than CMR. LVEF and GLS were similar in 2DEcho, 3DEcho and CMR. Patients with CMR‐determined RV free wall strain ≤ −14% tended to have lower peak VO_2_.

**Conclusions:**

Although 3DEcho underestimated RV volumes, it successfully identified all patients with RV dilatation based on method‐specific reference values.

## 1 BACKGROUND

The right ventricle (RV) plays an important role in the development of many cardiovascular diseases (Polak et al., 1983) (de Groote et al., 1998) (Haddad et al., 2008) and has prognostic value in patients with heart disease (Baumgartner et al., 2010) (Morris et al., 2016). However, the assessment of RV function is challenging, especially after repair of complex congenital defects such as tetralogy of Fallot, (ToF) with rapidly changing haemodynamics post‐surgery. A multitude of RV measurements has been proposed, but there is no universally accepted echocardiographic method to quantify RV function why cardiac magnetic resonance (CMR) frequently is used (Di Salvo et al., 2018). Conventional two‐dimensional echocardiography (2DEcho) produces basic quantification (Rudski et al., 2010) (Lang et al., 2015) (Simpson et al., 2016) but 2DEcho parameters have limitations, for example, due to the influence of afterload on measures of systolic function and the underestimation of tissue velocity due to the angle of insonation. Moreover, functional measurements based on a 5 mm × 5 mm region of interest do not represent the function of the entire chamber. The ability of longitudinal strain to indicate RV contractile dysfunction has been discussed in recent recommendations (Morris et al., 2016), which made exercise capacity interesting to relate to peak RV global strain.

We aimed to study the agreement between three‐dimensional echocardiography (3DEcho) and CMR for RV volumes and ejection fraction in ToF and after repair of pulmonary stenosis, and between 2DEcho, 3DEcho and CMR for RV strain. We also studied the possibility to identify enlarged RVs with 3DEcho compared to CMR. Furthermore, we compared the exercise capacity of the patients to their strain and volume measurements to evaluate if functional limitation could be related to the degree of RV dysfunction.

## 2 METHODS

### 2.1 Patient population

**FIGURE 1:**
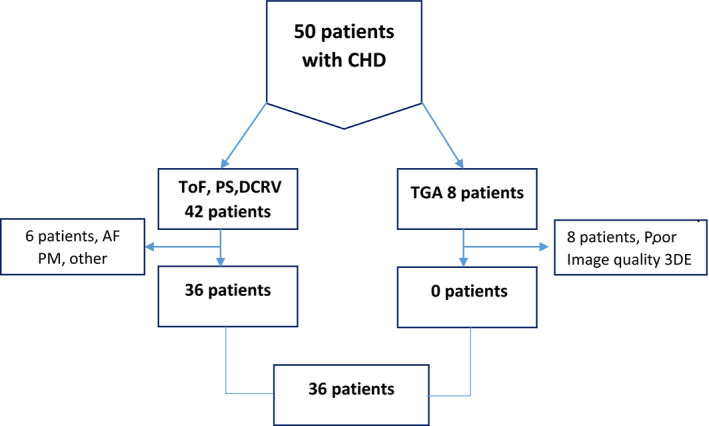
Flow chart. PS, pulmonary stenosis; ToF, tetralogy of Fallot; DCRV, double‐chamber right ventricle

50 patients that had undergone surgical treatment for congenital anomalies, followed‐up at the outpatient clinic of the Department of Cardiology at Linkoping University Hospital, were screened for participation in the study. Eight patients after Mustard corrected transposition of the great arteries were not included due to poor image quality on 3DEcho, 2 patients due to malignancy and 4 patients due to atrial arrythmia. Finally, 36 patients were included, see flow chart in Figure 1 and further patient characteristics in Table 1.

**Table 1:** Patient characteristics

Variable	Patients *N* 36 (±*SD*)
Age years	36 (±4)
BMI kg/m^2^	24 (±4)
BSA m^2^	1.9 (±0.25)
SBP mmHg	116 (±14)
DBP mmHg	74 (±12)
RV sys pressure mmHg	20 (±16)
Heart rate beats/min	71 (±13)
QRS duration msec	139 (±29)
NYHA I/II/III/IV (%)	47/47/6/0

Abbreviations: BMI, Body mass index; BSA, Body surface area; DBP, diastolic blood pressure; NYHA FC, New York Heart Association Functional Classification; RV, Right Ventricle; SBP, systolic blood pressure; *SD*, Standard deviation.

Twenty‐nine participants had corrected TOF, five pulmonary valvulotomy because of pulmonary valve stenosis (PS), and 2 had received correction for double‐chamber RV (DCRV). The surgical corrections were trans‐ventricular in 20 patients, trans‐annular in 12, and trans‐atrial in 2. Twenty‐one patients had surgery once, 9 twice, 4 three times and 1 patient had surgery 4 times. Two of the patients had undergone balloon dilatations and 14 haft pulmonary homograft replacements (PVR). The mean age at the time of the first surgical correction was 4 years (0–20 years). The mean time between the last surgery and inclusion was 20 ± 14.8 years. At the time of the investigation, 11 patients had more than moderate pulmonary regurgitation and only 4 had pulmonary stenosis with peak systolic velocity > 2.5 m/s. Each participant underwent 3D and 2DEcho, cardiopulmonary exercise testing (CPET) and CMR, all within a period of 4 hr. 3D images were acquired with and without a contrast agent to study the effects of contrast on myocardial delineation (SonoVue, Bracco Imaging S.p.A. Italy).

### 2.2 Ethics

The regional Ethical Review Board in Linköping Sweden approved the study protocol (Registration number 2012/334‐31). The study was performed in accordance with the Declaration of Helsinki. All participants gave written informed consent for inclusion in the study. The study is registered in the ISRCTN registry with the number 18376089.

### 2.3 Acquisition of 2DEchocardiography

A complete echocardiographic study in 2D and tissue Doppler imaging mode for the assessment of left ventricular (LV) and RV function was performed according to current guidelines (Rudski et al., 2010) (Mertens & Friedberg, 2010). Ultrasound data were acquired using a Vivid 9 scanner (GE Healthcare) with a 3.5 MHz M5S ultrasound probe for 2DEcho and with a 4V‐D transducer for the acquisition of 3D images. Measurements and semi‐automatic strain analysis were performed offline.

### 2.4 Analysis of 2DEchocardiography

**FIGURE 2:**
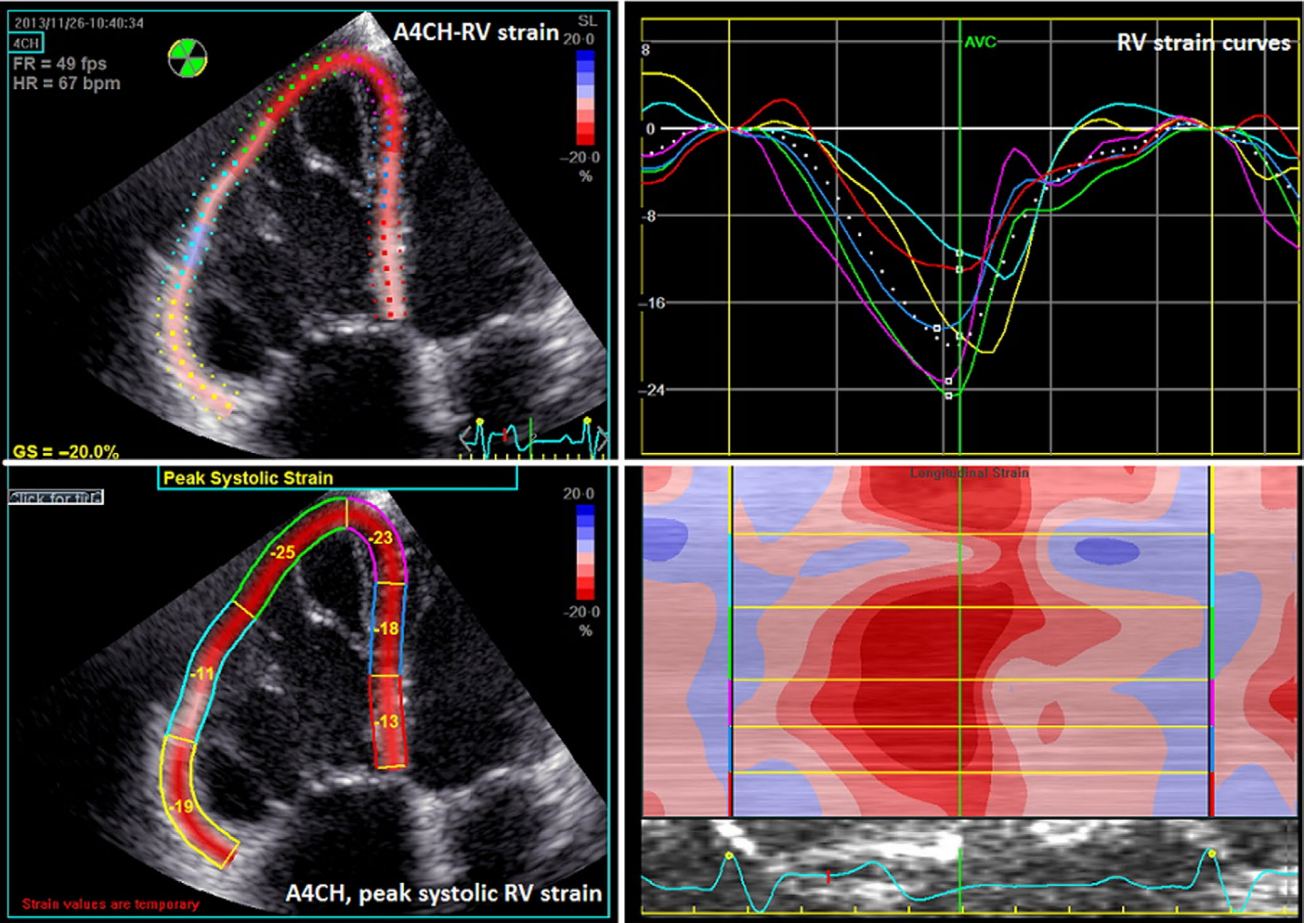
Longitudinal echocardiographic strain analysis of the right ventricle. Longitudinal Strain analysis of the right ventricle (RV). In the first step, the user sets the points in the posterior and septal RV wall at the level of the tricuspid valve and in the RV apex starting the tracking of the RV endocardial border in the apical four‐chamber view (A4CH) (left top). The strain curves are generated by the software (right top). After approval of the endocardial tracking by the user, peak systolic strain values are displayed for all six RV segments and for global systolic RV strain (left bottom). AVC, aortic valve closure

Analysis of stored files was performed using commercially available software (EchoPac BT 13, GE Healthcare). LV volumes were calculated according to the biplane Simpson method. To measure RV global longitudinal strain (RVGLS), we manually traced the RV endocardial border in the four‐chamber (4CH) view using the 2D‐strain function of Echopac. The segmental tracking quality was assessed visually and if necessary corrected manually. The software then automatically generated strain curves for each RV segment. Peak RV GLS was calculated by averaging the values obtained from all RV segments. RV free wall longitudinal strain (FWLS) was calculated by averaging the strain values of the three lateral wall segments, Figure 2.

### 2.5 Acquisition of 3DEchocardiography

Images were obtained from a modified RV‐focused apical view (Lang et al., 2012). Special effort was made to include the whole lateral RV wall and the LV apex in the image sector. To acquire 3D full‐volume data sets, four to six electrocardiographically gated cardiac cycles were obtained during breath hold. The data sets were stored in DICOM (Digital Imaging and Communications in Medicine) format. We did not acquire 3D volumes of the LV. Two observers blinded to patient‐related information independently measured RV volumes, with observer 2 only providing results for measurements of reproducibility. We analysed the data using 4D‐RV‐Function 2.0 (TOMTEC Imaging systemv, GmbH, Unterschleissheim, Germany) as a plugin to the Echopac software.

### 2.6 Analysis of 3DEchocardiography

**FIGURE 3:**
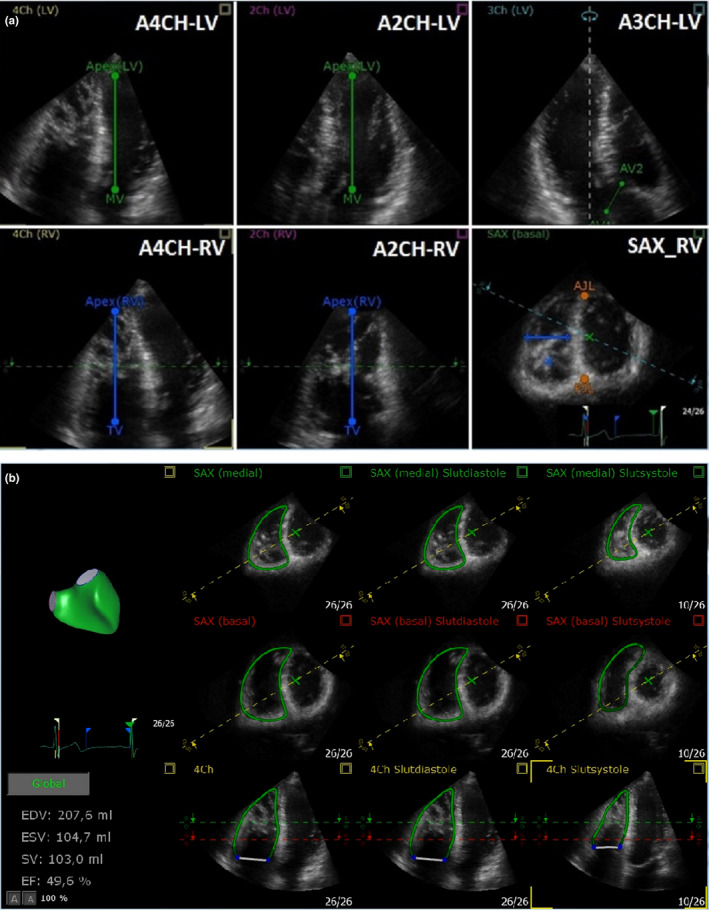
(a) Three‐dimensional echocardiographic analysis of the right ventricle. Three‐dimensional analysis of the right ventricle (RV), orientation and anatomic landmarks. In the first step, the user sets the left (top) and right (bottom) ventricular long axes in the apical four‐chamber view (A4CH) (A), apical two‐chamber view (A2CH) (B) at end‐diastole and the left ventricular outflow tract (LVOT) diameter is marked in the apical three chamber (A3CH) view (C, top). Than anterior (AJL, Anterior junction line) and posterior (PJL, posterior junction line) junctions of the right ventricular free wall with the interventricular septum, as well as the longest dimension of the right ventricular cavity between the septum and the free wall are set, both in a single short‐axis (SAX) view (C, bottom). AV aortic valve annulus; LV left ventricle; MV mitral valve; PJL posterior junction line; AJL anterior junction; TV tricuspid valve. (b) Three‐dimensional echocardiographic analysis of RV volume with TOMTEC. The software algorithm analyses the data and adapts the 3D model based on input information from the position of the requested anatomical landmarks. EDV, end‐diastolic volume; ESV, end‐systolic volume; SV, slag volume; EF ejection fraction; the white line represents tricuspid valve, 4Ch, four‐chamber view, SAX, short‐axis view

We judged the 3DEcho image quality on a scale from 1 to 4 (1 = very good, 4 = poor image quality). The addition of ultrasound contrast did not improve on the measurements and is not further discussed here (van den Bosch et al., 2004). We performed 4D RV analysis in several steps that defined the long and short axes of the RV and the insertion points of the RV on the interventricular septum, Figure 3a.

The 4D‐RV‐Function 2.0 algorithm creates a 3D model (“beutel”) of the RV based on anatomical landmarks. The operator can adjust the model manually by moving the contour of the RV endocardium, Figure 3b (Gopal et al., 2007). In the last step, the software used the model data set to calculate RVGLS, RVFWLS, TAPSE and FAC. The results of observer 1 were used for final results while the output of observer 2 was only used for calculating reproducibility.

### 2.7 CMR acquisition protocol and analysis

Cardiac magnetic resonance was performed on a 1.5 T scanner (Achieva Nova Dual, Philips Healthcare) equipped with a cardiac phased‐array receiver coil. Cine images (bSSFP) were acquired in 30 time frames during end‐expiratory breath‐hold employing retrospective electrocardiogram gating in the long‐axis two‐chamber (2CH), three chamber (3CH) and 4CH views of the LV, and also in a stack of SAX slices covering both ventricles from the base to the apex. The short‐axis stack had a slice thickness of 8 mm. Analysis was performed on a workstation equipped with the Segment v2.0 R5024 semi‐automatic software for cardiac volumetric analysis (Heiberg et al., 2010). RV atrioventricular plane displacement (AVPD) was assessed, by an automatic tracking algorithm implemented in the Segment v2.0 R5024 software (Seemann et al., 2017). This algorithm is based on tracking of the LV AV plane in the 4CH, 2CH and 3CH views and the RV AV plane in the PLAX, SAX and 4 CH views. Volumes and EF for both ventricles were derived from SAX slices after the endocardial borders were manually segmented, excluding the papillary muscles.

We used feature‐tracking software (2D‐Cardiac Performance Analysis‐MR v1.2, TOMTEC Imaging Systems GmbH) to calculate global and regional longitudinal strain for both ventricles. The software uses optical flow technology (Dobrovie et al., 2019) and calculated GLS by averaging the values of six segments in the LV and in the respectively, obtained in the 4CH view. Because it is at times conceptually difficult to grasp negative strain values, we used absolute numbers to describe longitudinal RV strain, denoting −22% longitudinal strain as a “greater” strain value than −20% longitudinal strain, according to the EACVI consensus document regarding standardized deformation imaging (Voigt et al., 2015).

### 2.8 Cardiopulmonary exercise test (CPET)

The study participants performed a maximum symptom‐limited (Borg scale of perceived exertion ≥ 17) CPET in a seated position on a cycle ergometer (Monark Ergomedic 839E, Monark Exercise AB, Vansbro, Sweden). Depending on the expected individual physical work capacity, cycling began with 5 min of steady‐state work at a load of 30 W or 50 W, followed by load increases in increments of either 10 W/min or 20 W/min with the goal of reaching maximal exercise capacity within 8–12 min. Respiratory gases were collected using an oral mask and analysed breath‐by‐breath using a Jaeger Oxycon Pro (Vyaire Inc). We calculated peak VO_2_ using the values measured during the last 60 s of exercise and expressed the results in terms of ml × kg^−1^ and ml × kg^‐1^ × min^−1^. We considered the participants to have achieved maximal exercise capacity if the respiratory exchange ratio was >1 continuously for 3 min or longer. We monitored the participants by continuous 12‐lead electrocardiogram and measured their blood pressure using a manual cuff manometer at rest and at 3‐min intervals during exercise. All patients underwent a spirometry test immediately before the CPET to obtain forced vital capacity and forced expiratory volume in 1 s measurements.

### 2.9 Reproducibility

We assessed the reproducibility of the 3DEcho and CMR measurements in all but one of the participants. Observer 1 and observer 2 assessed 3D‐based RV volumes, 3 months after the first measurements were taken. Observers 1 and 2 are both senior echocardiographers with more than 10 years of experience each. Variability of the CMR volume measurements was assessed by observer 1 and observer 3 in 15 randomly selected participants and variability of CMR‐FT strain by observer 1 and observer 4. Reproducibility for 2DEcho volumes was performed on all participants by observer 1 and 2.

### 2.10 Statistical analysis

We presented normally distributed, continuous variables as mean ± 1 standard deviation (*SD*) and range when appropriate. We used Pearson correlation coefficient and regression analysis to compare RV measurements derived from 3D and 2DEcho with the corresponding CMR values. The agreement between the methods and interobserver agreement were assessed with ICC and Bland–Altman plots.

We used paired *t* tests to assess the significance of differences between the measurements derived from CMR and those derived from 3DEcho. We performed all analyses using SPSS 23.0 (IBM SPSS Statistics). We considered differences among variables to be significant if *p* < .05.

## 3 RESULTS

### 3.1 2DEcho basal measurements

The average 2DEcho parameters among all participants were as follows: LVEDV 109 ± 26 ml LVESV 50 ± 13 ml, LVEF 55% ± 6.

### 3.2 Comparison of volumes and ejection fraction measured with 3DEcho and CMR

We obtained RV volumes by 3DEcho in 35 (97%) of the participants. The 3DEcho image quality was in the two highest categories for 26 (74%) of the participants. The mean values for RV (assessed with 3DEcho and CMR) and LV volumes (assessed with CMR and 2DEcho) are presented in Table 2. CMR assessed RV volumes were significantly greater than the LV volumes [difference in EDV 42 ml, *p* < .001; difference in ESV 35 ml, *p* < .001]. The RVEF was lower than the LVEF; difference −6%, *p* < .001.

**Table 2:** Volumes, dimensions and functional measurements

Parameter	CMR ± *SD*	3DEcho ± *SD*	2DEcho ± *SD*
RVEF% Min–max	43 (±8) 23–60	46 (±8) 27–61	na
FAC% Min–max	na	39 (±7) 22–50	44 (±8) 30–58
RVEDV ml Min–max	197 (±59) 111–339	188 (±53) 115–322	na
RVEDVi ml/m^2^ Min–max	106 (±24) 65–163	102 (±25) 67–184	na
RVESV ml Min–max	114 (±41) 62–260	100 (±30) 52–175	na
RVESVi ml/m^2^ Min–max	61 (±18) 34–111	54 (±13) 35–88	na
LVEDV ml Min–max	156(±31) 107–222	na	109 (±26) 66–177
LVESV ml Min‐max	80 (±18) 51–122	na	50 (±13) 28–83
LVEDVi ml/m^2^ Min‐max	84 (±13) 61–122	na	57 (±32) ml/m^2^
LVEF % Min–max	49 (±6) 37–60	na	55 (±6) 42–67
RVAVPD mm	11 (±3)	na	na
LVAVPD mm	10 (±1)	na	na
TAPSE mm Min‐max	na	15 (±5) 4–25	15 (±3) 9–21
RVGLS % Min–max	19 (±6) 11–36	na	17 (±3) 12–25
RVFWLS % min–max	18 (±8) 8–43	21 (±6) 10–32	17 (±4) 5–28
RV S′ cm/s Min–max	na	na	8 (±3) 4–14

Abbreviations: 2D, two‐dimensional; 3D, three‐dimensional; AVPD, atrioventricular plan displacement; CMR, Cardiac Magnetic Resonance; EDV, end‐diastolic volume; EF, ejection fraction; ESV, end‐systolic volume; FWLS, free wall longitudinal strain; GLS, global longitudinal strain; i, indexed; LV, Left ventricle; na, not available; RV, Right ventricle; S′ RV, lateral wall velocity; *SD*, standard deviation; TAPSE, tricuspid annular plane systolic excursion.

### 3.3 RV and LV myocardial deformation

The CMR‐determined RVGLS −19.5 ± 6% was higher than that determined by 3DEcho ‐ −17.6 ± 4% and 2DEcho −17 ± 4%. The highest values of RVFWLS were those based on 3DEcho −21% ± 5%; (CMR: −18.5 ± 8%; 2DEcho: −16.5 ± 3%).

The LVGLS was −20% ± 4% in males and −22.6% ± 5.3% in females, at the level previously reported in TOF population (Kempny et al., 2012).

### 3.4 RV and LV atrioventricular plane displacement by CMR

RVAVPD measured by CMR was 9.8 ± 2.4 mm in males and 11.4 ± 2.5 mm in females. The r‐squared values for the correlations between TAPSE by 3D and 2DEcho and RVAVPD measured by CMR were *r* ^2^ = .37 and *r* ^2^ = .35, respectively, (*r* = .06 for both).

LVAVPD measured by CMR was 10.4 ± 1.4 mm in males and 10.9 ± 1.8 mm in females—at the lower limit of normal according to the reference values (9 mm and 11 mm, respectively).

### 3.5 Cardiopulmonary exercise capacity in relation to RV longitudinal strain

Females had lower average exercise capacity than males of 126 ± 36 W and peak VO_2_ = 25 ± 5 ml × kg^−1^ × min^−1^, while males had 205 ± 55 W and peak VO_2_ = 29 ml × kg^−1^ × min^−1^. Five of 17 females and 10 of 19 males with RVFWLS strain by CMR lower than −14% had depressed exercise capacity in comparison with patients with a strain higher than −14%, Table 3. However, no correlation was found between RV volumes and RVFWLS, and no correlation between RV volumes and exercise capacity.

**Table 3:** CMR‐FT strain and exercise capacity

RVFWLS > −14%		
Parameter	Female *N* = 12	Male *N* = 9
	Mean ± *SD* [CI]	Mean ± *SD* [CI]
CPET Watt max	138 ± 33 [73; 203]	227 ± 54 [121; 332]
CPET VO_2_ ml × kg^−1^ × min^−1^	26 ± 4 [18; 34]	32 ± 8 [16; 48]
TAPSE 3DEcho	17 ± 5 [7; 27]	14 ± 6 [2; 26]
TAPSE 2DEcho	16 ± 4 [9; 23]	14 ± 4 [7; 21]

RVFWLS ≤ −14%		
Parameter	Female *N* = 5	Male *N* = 10
	Mean ± *SD* [CI]	Mean ± *SD* [CI]
CPET Wat max	96 ± 26 [43; 149]	186 ± 51 [86; 286]
CPET VO_2_ ml × kg^−1^ × min^−1^	22 ± 5 [12; 33]	27 ± 8 [12; 42]
TAPSE 3DEcho	14 ± 3 [9; 19]	12 ± 4 [5; 20]
TAPSE 2DEcho	14 ± 1 [12; 17]	13 ± 2 [8; 18]

Abbreviations: CI, Confidence Interval; CPET, Cardiopulmonary exercise test; RVFWLS, Right ventricular free wall longitudinal strain; *SD*, standard deviation; TAPSE, Tricuspid annular plane systolic excursion.

### 3.6 Correlations between 3DEcho and CMR

**FIGURE 4:**
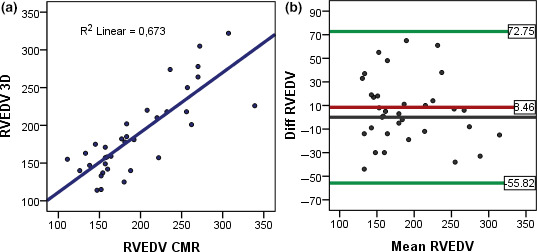
(a) Linear regression, correlation between RVEDV measured by CMR and 3DEcho. (b) Bland–Altman plots, agreement between CMR and 3DEcho for RVEDV

**FIGURE 5:**
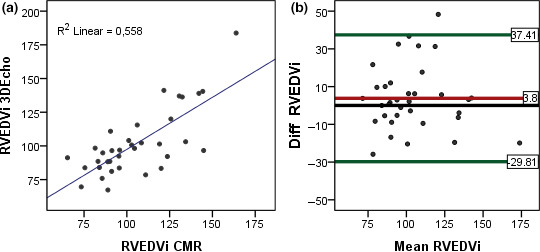
(a) Linear regression, correlation between RVEDVi measured by CMR and 3DEcho. (b) Bland–Altman plots, agreement between CMR and 3D Echo for RVEDVi

The 3DEcho measurements of RVEDV and EDV‐indexed to BSA showed moderate correlations with the CMR volumes (*r* = .82 and *r* = .75, respectively). Bias was low, but the limits of agreement were large, similar for low volumes as well as high volumes, Figures 4a,b and 5a,b.

### 3.7 Reproducibility

**FIGURE 6:**
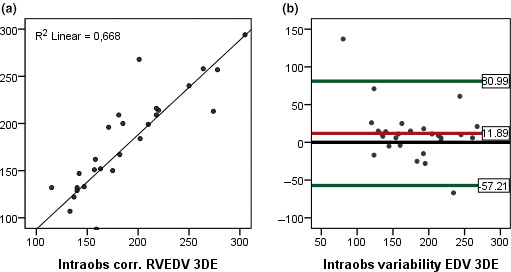
(a) Linear regression, intraobserver correlation, for RVEDV measured with 3DEcho. (b) Bland–Altman plots, intraobserver variability for RVEDV measured with 3DEcho

Measured with 3DEcho, the interobserver variability (ICC) for RVEDV was 0.68 and for ESV = 0.67. Bias and limits of agreement are depicted in Figure 6a,b. ICC for 2DEcho LVEDV was 0.62.

Measured with CMR, the interobserver variability (ICC) for RVEDV was 0.96, for ESV = 0.97 and for EF = 0.78. Interobserver variability for CMR‐FT assessed RV strain was low with ICC = 0.94.

**FIGURE 7:**
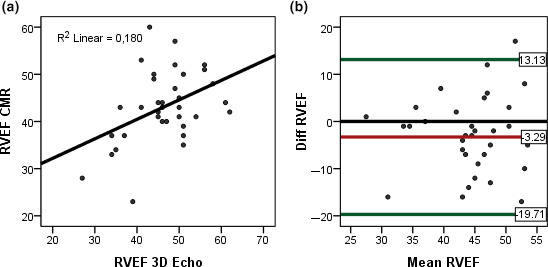
(a) Linear regression for RVEF between 3DEcho and CMR. (b) Bland–Altman plots, agreement between CMR and 3D Echo for RVEF

**FIGURE 8:**
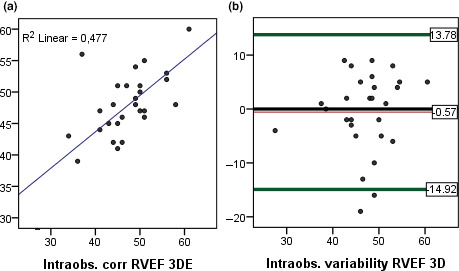
(a) Linear regression, intraobserver correlation for RVEF with 3DEcho. (b) Bland–Altman plots, intraobserver variability for RVEF measured with 3DEcho. 3DEcho Three‐dimensional echocardiography, RV, Right ventricle, EDV, End‐diastolic volume, EDVi, End‐diastolic volume indexed, EF, ejection fraction, CMR, Cardiac Magnetic Resonance

The intraobserver variability with 3DEcho was higher for RVEDV (ICC = 0.89) than for RVESV (ICC = 0.94). The correlation between CMR and 3DEcho for RVEF as well as bias and limits of agreement for intraobserver variability with 3DEcho for RVEF is depicted in Figures 7a,b and 8a,b.

Interobserver variability ICC for LV myocardial strain measured with 2DEcho speckle tracking was 0.83.

## 4 DISCUSSION

We showed that most patients with corrected ToF had RV dilatation as determined from CMR as well as 3DEcho. The RV volumes were generally larger than the corresponding reference values for the methods (3DEcho and CMR) by gender (Kawel‐Boehm et al., 2015; Tamborini et al., 2010) and larger for CMR than for 3DEcho (11% for EDV and 17% for ESV). In comparison with the reference values for LV volumes obtained with CMR (Kawel‐Boehm et al., 2015), the study participants had higher ESV and lower EF (48 ± 6.5% in males and 50 ± 4.8% in females), while EDV was within normal limits.

We investigated whether 3DEcho could identify patients with RV dilatation based on reference values for 3DEcho (Tamborini et al., 2010). Comparison of the 3DEcho measurements with the 3DEcho reference values indicated that 95% of the males and 100% of the females had RV dilatation. By contrast, comparison with the CMR reference values (Kawel‐Boehm et al., 2015) indicated that 68% of the males and 94% of the females had RV dilatation. Thus, in comparison with CMR, 3DEcho overestimated RVEDV in 6 of 35 patients. Both 2D and 3DEcho could serve as screening methods for the detection of abnormal RV function (Lang et al., 2015; Rudski et al., 2010). Our patients reflect the previous and current practice of surgical palliation of congenital heart defects in Sweden. The 2DEcho scans showed that TAPSE and S´ of the RV free wall were below the lower limit of normal, while FAC for the RV was within the normal range according to current guidelines (Rudski et al., 2010), RVGLS and RVFWLS were both lower than −20%, indicating RV dysfunction (Lang et al., 2015; Morris et al., 2017; Muraru et al., 2016).

We found moderate correlations between RV volumes and RVAVPD. The correlations between AVPD measurements and other parameters of RV lateral deformation were sufficiently strong to allow 2D and 3D measurements of TAPSE to be used interchangeably in the follow‐up of patients with TOF, according to guidelines for RV functional assessment (Rudski et al., 2010). We compared three methods for the measurement of RVFWLS. Two methods (CMR and 2DEcho) showed RV functional impairment according to guidelines and published papers (Lang et al., 2015; Liu et al., 2018; Muraru et al., 2016), but vendors have different reference values concerning strain, which means that strain measurements produced by the different methods are not interchangeable (Barreiro‐Perez et al., 2018). To our knowledge, there are not yet any reference values for RV strain assessment by 3DEcho. In comparison with the 3DEcho and CMR reference values (Kawel‐Boehm et al., 2015), our study population had as expected greater RV volumes, because of prior surgical treatment and remaining abnormalities such as pulmonary stenosis or regurgitation. In the study by Medvedofsky, the RV volumes in patients were similar to those in our study, but the agreement between CMR and 3DEcho was higher (Medvedofsky et al., 2015). That can be explained by the fact that the patients in the Medvedofsky study had normal RV anatomy. The lower intertechnique agreement in our study suggests that the size of the RV and a history of cardiac surgery impair 3D endocardial tracking, possibly due to the presence of hypertrophied trabeculae and papillary muscles in TOF (Xu et al., 2014). The difference between volumes measured by CMR and 3DEcho also relates to the different algorithms used. CMR uses disc summation, which is difficult to apply in the tricuspid valve plane and RVOT which could increase measurement variability. On the other hand, the 3DEcho software may not have been trained to detect an altered RV anatomy after thoracic surgery, which makes it less than optimal for the patients in our postsurgical study population (Crean et al., 2011).

## 5 CONCLUSION

We found that RV dilatation according to method‐specific reference values was more common when investigated with 3DEcho than with CMR. We also demonstrated that patients with RVFWLS by CMR lower than −14% tended to display depressed exercise capacity. Our observations indicate a need for further studies concerning the relative merits of different modalities for the follow‐up of patients with RV dilatation.

### 5.1 Limitations

Several factors need to be considered before our findings can be extended to patients with congenital heart disease in general. Our study population was small and limited to patients with corrected TOF and PS. Patients with TGA were not included in this study because of relatively poor image quality and difficulties in the identification of anatomic landmarks. Severely distorted anatomies are currently too difficult to scan with 3DEcho. Furthermore, the 3DEcho software may not have been developed with an eye on particular anatomical distortions. There are relatively few reference values for 3DEcho, because the technology is still in an early stage of development. For the same reason, our staff had relatively little prior experience scanning patients with 3DEcho and it has been shown that reproducibility of measurements increases with increasing observer experience. The interobserver variability for 3DEcho was high, which limits the feasibility of the method. When measuring RVAVPD with CMR, we used the average of three points on the tricuspid annulus and not the single measurement used by Kawel‐Boehm.

## CONFLICT OF INTEREST

The authors have no conflicts of interest.
